# Antioxidant Biomarkers from *Vanda coerulea* Stems Reduce Irradiated HaCaT PGE-2 Production as a Result of COX-2 Inhibition

**DOI:** 10.1371/journal.pone.0013713

**Published:** 2010-10-28

**Authors:** Charlotte Simmler, Cyril Antheaume, Annelise Lobstein

**Affiliations:** 1 Faculty of Pharmacy, University of Strasbourg, Illkirch, France; 2 Laboratoire de Pharmacognosie (UMR-CNRS 7200), Illkirch, France; 3 Service Commun d'Analyse (SCA)-RMN, Illkirch, France; Tufts University, United States of America

## Abstract

**Background:**

In our investigations towards the isolation of potentially biologically active constituents from Orchidaceae, we carried out phytochemical and biological analyses of *Vanda* species. A preliminary biological screening revealed that *Vanda coerulea* (Griff. ex. Lindl) crude hydro-alcoholic stem extract displayed the best DPPH /^•^OH radical scavenging activity and *in vitro* inhibition of type 2 prostaglandin (PGE-2) release from UV_B_ (60 mJ/cm^2^) irradiated HaCaT keratinocytes.

**Principal Findings:**

Bio-guided fractionation and phytochemical analysis led to the isolation of five stilbenoids: imbricatin (**1**) methoxycoelonin (**2**) gigantol (**3**) flavidin (**4**) and coelonin (**5**). Stilbenoids (**1**–**3**) were the most concentrated in crude hydro-alcoholic stem extract and were considered as *Vanda coerulea* stem biomarkers. Dihydro-phenanthropyran (**1**) and dihydro-phenanthrene (**2**) displayed the best DPPH/^•^OH radical scavenging activities as well as HaCaT intracellular antioxidant properties (using DCFH-DA probe: IC_50_ 8.8 µM and 9.4 µM, respectively) compared to bibenzyle (**3**) (IC_50_ 20.6 µM). In turn, the latter showed a constant inhibition of PGE-2 production, stronger than stilbenoids (**1**) and (**2**) (IC_50_ 12.2 µM and 19.3 µM, respectively). Western blot analysis revealed that stilbenoids (**1**–**3**) inhibited COX-2 expression at 23 µM. Interestingly, stilbenoids (**1**) and (**2**) but not (**3**) were able to inhibit human recombinant COX-2 activity.

**Conclusions:**

Major antioxidant stilbenoids (**1**–**3**) from *Vanda coerulea* stems displayed an inhibition of UV_B_-induced COX-2 expression. Imbricatin (**1**) and methoxycoelonin (**2**) were also able to inhibit COX-2 activity in a concentration-dependent manner thereby reducing PGE-2 production from irradiated HaCaT cells. Our studies suggest that stilbenoids (**1**–**3**) could be potentially used for skin protection against the damage caused by UV_B_ exposure.

## Introduction

Skin is continuously exposed to ultraviolet (UV) radiations from sunlight and to environmental pollution. UV_B_ (290–320 nm) radiations trigger the production of reactive oxygen species (ROS), increasing oxidative stress in irradiated skin. ROS are an inherent part of the anabolism and catabolism of tissues, including skin. Cellular enzymes and controlled metabolic processes ordinarily keep oxidative damage to cells at a minimum. In times of increased ROS production (caused by sunlight, smoke and pollution) protective controls may not be adequate anymore resulting in an oxidant-antioxidant imbalance, defined as oxidative stress. It can cause many adverse effect (ageing) and pathological conditions (inflammation, cancer) via the modulation of biochemical, genetic and signal transduction pathways [1]–[3].

UV_B_ radiations induce cyclo-oxygenase 2 (COX-2) expression and subsequently increase the production of prostaglandin E2 (PGE-2) in keratinocytes. COX-2 is a rate limiting enzyme for the generation of prostaglandin metabolites and its expression has been linked to the pathophysiology of inflammation and cancer [4]. PGE-2, the major prostaglandin synthesized by COX-2, is produced abundantly by keratinocytes under UVs exposure, especially in aged human skin [5]. This prostaglandin is known to play an important role in skin inflammation as it is responsible for erythema, edema and vascular permeability facilitating the infiltration of neutrophils to the dermis [6]. It also increases keratinocytes proliferation and differentiation and is consequently involved in epidermal homeostasis and repair [7]. Finally, UV_B_ increase oxidative stress and the generated reactive oxygen intermediate are most likely involved in the up-regulation of COX-2 [7], [8]. Consequently, both ROS and PGE-2 are critical mediators of skin inflammation process. Inhibition of COX-2 enzyme is considered as an important mechanism for skin photoprotection [9].

Plants have also to protect themselves from solar radiation. Thus, they synthetize natural chromophores like polyphenolic compounds (flavonoids, stilbenoids and tannins) that are able to absorb UV radiations and display antioxidant properties [10]. Therefore, there has been considerable interest in the use of natural occuring plant products for the prevention of UV-induced skin damages. Polyphenols possessing skin anti-inflammatory and antioxidant properties are among the most promising group of natural compounds that can be exploited as ideal photoprotective agents [11]. Recently, polyphenols such as silymarine from milk thistle, soy isoflavones including genistein, green tea catechins, proanthocyanidins and resveratrol from grape seeds, have been demonstrated to have potent photoprotective properties when used topically on skin. These antioxidant polyphenols were all able to reduce UV_B_-induced inflammatory responses, partly due to the suppression of COX-2 expression and/or its enzymatic activity [10]–[12].

**Figure 1:**
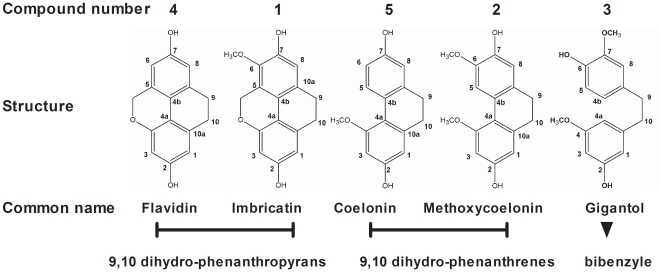
Structures of stilbenoids isolated from *V. coerulea* stems. Stilbenoids isolated from *Vanda coerulea* stems belong to the phenanthrene group including 9,10 dihydro-phenanthrenes, 9,10 dihydro-phenanthropyrans and bibenzyles. Carbone numbering relative to NMR data is represented here. Compounds 1 and 4 are 9,10 dihydro-phenanthropyrans, compounds 2 and 5 are 9,10 dihydro-phenanthrenes and compound 3 is a bibenzyle.

The Orchidaceae is subdived in five subfamilies. Genera form the Epidendroideae subfamily such as *Dendrobium*, *Bletilla*, *Cymbidium*, *Gastrodia* and *Vanda* occurred mostly in tropical areas [13]–[14]. Flavones *C*-glycosides [15], hydroxybenzyle derivatives [16], fluorenones and stilbenoids [17]–[20] are the most commun polyphenolic compounds described for these orchids. Many cosmetic patents protected the use of different orchid extracts but without any information about the identity of their active compounds. Orchidaceae from the genus *Vanda* have not been well studied considering their biological activities on human cells and their chemical composition. Only two kinds of phenanthropyran have been described in the species *V. parviflora* [21] and *V. tesselata* [22], which has been demonstrated to display wound healing properties [23]. These observations led us to focus our interest to *Vanda coerulea* Griff. ex Lindl, also known as the “Blue Orchid”. A preliminary biological screening on stem, root and leaf crude hydro-alcoholic extracts revealed that *V. coerulea* stem extract displayed best DPPH /OH radical scavenging activity and *in vitro* inhibition of PGE-2 production from irradiated HaCaT (genetically modified keratinocytes). Bio-guided fractionation combined with phytochemical analysis allowed us to isolate and identify five stilbenoids (**Figure 1**): two 9,10 dihydro-phenanthropyrans imbricatin (**1**), and flavidin (**4**), two 9,10 dihydro-phenanthrenes methoxycoelonin (**2**) and coelonin (**5**) and one bibenzyle gigantol (**3**). Additional compounds identified were carbohydrates and terpenoids such as sesquiterpenes and phytosterols.

In the present work, we examine whether stilbenoids from *Vanda coerulea* stems display radical scavenging activities, attenuate intracellular ROS formation and reduce both UV_B_-induced PGE-2 production on HaCaT cells and COX-2 activity. Comparison of their radical scavenging properties led us to a first structure-activity relation (SAR) study performed with orchid's stilbenoids. Biological analyses on HaCaT cells were performed with the three most concentrated stilbenoids (**1**–**3**). We evaluated here for the first time the potential interest of stilbenoids from Orchidaceae as new skin photoprotecting agents.

## Materials and Methods

### Reagents

Quercetin was obtained from Extrasynthese. Luminol was obtained from Fluka Biochemika. Indomethacin, tocopherol and all other chemicals like dimethylsulfoxyde (DMSO), 2′,7′-dichlorodihydrofluorescein diacetate (DCFH DA) probe, were from Sigma. Keratinocytes serum free medium (KSFM), foetal bovine serum (FBS) for HaCaT culture, penicillin/streptomycin, glutamine and phosphate buffer serum (PBS) were from Invitrogen. Cell proliferation kit II reagent (XTT) was provided by Roche Diagnostic. Bicinchoninic acid (BCA) assay kit for protein dosage was obtained from Uptima Interchim. Enzyme immunoassay (EIA) kits for PGE-2 were obtained from R&D Systems and Cayman Chemical. Antibodies anti β-actin (sc-130300) and anti COX-2 (sc-19999) as well as CCD-1064SK cell lysate were provided by Santa Cruz Biotechnology Inc. Amersham Hybond ECL nitrocellulose membrane, horseradish peroxidase-conjugated secondary antibody (NIF-825) and enhanced chemiluminescence (ECL) kit were obtained from GE Healthcare Europe GmbH.

### Plant material

*Vanda coerulea* Griff. ex Lindl. was identified by Dr. Josef Margraf in Thailand. A voucher specimen (No HITBC128192) was deposited in the Herbarium of the Xishuangbanna Tropical Botanical Garden (XTBG). Stems from flowering specimens were collected in October 2006. Cut stems were dried by sunshine on hanging basket to avoid contamination by soil and prevent fungus development. After reception in France, dried cut stems were finally reduced to powder in the ultra-centrifuge crusher (Retsch ZM 200) just before extraction. Supply of stems was carried out in accordance with CITES (Convention on International Trade in Endangered Species of wild Fauna and Flora) regulations.

### Extraction and isolation

Dried *V. coerulea* stems (100 g) were exhaustly extracted with EtOH-H_2_O (9∶1) under reflux and filtered to provide a red brown crude hydro-alcoholic extract (8.9 g). Dried stems (100 g) were also successively extracted by cyclohexane (C_6_H_12_), dichloromethane (CH_2_Cl_2_) and methanol (MeOH) using an automatisable Soxtec (Avanti 2055), to yield C_6_H_12_ (1.0 g), CH_2_Cl_2_ (1.0 g), and MeOH (5.8 g) extracts, respectively. An aliquot of CH_2_Cl_2_ extract (0.64 g), which concentrated stilbenoids, was suspended in MeOH-CH_2_Cl_2_ (8∶2) and subjected to Sephadex LH20 column (H: 27 cm d: 2.5 cm) with MeOH-CH_2_Cl_2_ (8∶2) elution and collection of 25 fractions of 10 mL. Fractions with similar TLC (Thin Layer Chromatography) profiles were gathered. Fractions 3 to 6, rich in stilbenoids, were further purified by semi preparative reversed-phase HPLC to give compounds **1** (70 mg), **2** (35 mg) **3** (19 mg), **4** (8 mg) and **5** (8 mg).

Extract and fractions were analyzed by RP-HPLC using a Nucleodur C18ec column (5 µm, 250 mm×4.6 mm i.d.) in a Varian chromatograph equipped with a Prostar 230 pump and a Prostar 330 diode-array detector. The column was eluted with MeOH-(H_2_O+0.1% HCO_2_H) using a linear gradient at a flow rate of 1 mL.min^−1^, starting from 20% up to 47% MeOH in 13.5 min, maintaining 47% MeOH during 10 min and finally from 47% to 100% MeOH in 10 min. The fractions were monitored between 200 and 400 nm. Semi-preparative HPLC purifications of stilbenoids were carried out on another chromatograph equipped with a Gilson 322 pump and a UV-VIS 151 Gilson wavelength detector, using a Nucleodur C18ec column (5 µm, 250 mm×21 mm i.d.) eluted with MeOH-(H_2_O+0.1% HCO_2_H) under the following conditions: 47% MeOH during 10 min and to 100% MeOH in 30 min at a flow rate of 15 mL.min^−1^. The fractions were monitored at 210 nm.

Structures of these stilbenoids were elucidated by spectral analyses (^1^H-NMR, ^13^C NMR, HMBC, HSQC, NOESY, and HR-ESI-MS) and confirmed by comparison with those reported in literature [24]–[29].

*Imbricatin* (**1**): *yellow gum,* UV (in MeOH) λ_max_ (log ξ) 217 (4.4), 285 (4), and 307 (4) nm characteristic of a dihydro-phenanthrene skeleton ^1^H NMR (CDCl_3_, 400 MHz): δ = 6.29 (1H, d, *J* = 2.5 Hz, H-1), 6.31 (d, 1H, *J* = 2.5 Hz, H-3), 6.73 (s, 1H, H-8), 2.79-2.78 (4H, d, *J* = 3.9 Hz, H-9 and H-10) and 5.19 (2H, s, --O--CH_2_--Ar), 3.77 (3H, s, H-OMe).^13^C NMR data (CDCl_3_, 100 MHz) δ = 108.7 (C-1), 155.0 (C-2), 101.8 (C-3), 120.1 (C-4a), 113.1 (C-4b), 153.0 (C-4), 121.0 (C-5), 141.0 (C-6), 147.6 (C-7), 114.4 (C-8), 129.8 (C-8a), 136.0 (C-10a), 28.0 (C-10), 27.5 (C-9) 63.8 (C- O--CH_2_--Ar), 62.2 (C-OMe); HR-ESI-MS ion [M-H]**^-^** at *m/z* = 269.0815 (Molecular formula calculated for C_16_H_14_O_4_:270.0892, difference 1.65 ppm).

*Methoxycoelonin* (**2**): *yellow brown gum,* UV (in MeOH) λ_max_ (log ξ) 216 (4.3), 285 (3.9), and 307 (3.8) nm characteristic of dihydro-phenanthrene skeleton ^1^H NMR (CDCl_3_, 400 MHz): δ = 6.30 (d, 1H, *J* = 2.5 Hz, H-1), 6.37 (1H, d, *J* = 2.5 Hz, H-3), 7.81 (1H, s, H-5), 6.72 (1H, s, H-8), 2.69 (4H, m, H-9 and H-10) and 3.86 (3H, s, C5-OMe), 3.82 (3H, s, C6-OMe ). For ^13^C NMR data (CDCl_3_, 100 MHz): δ =  107.8 (C-1), 154.1 (C-2), 97.8 (C-3), 124.3 (C-4a), 116.3 (C-4b), 156.9 (C-4), 110.8 (C-5), 143.6 (C-6), 142.9 (C-7), 112.7 (C-8), 130.9 (C-8a), 140.8 (C-10a), 28.6 (C-9), 30.4 (C-10), 55.3 (C-OMe), 54.9 (C-OMe)**;** HR-ESI-MS ion [M-H]**^-^** at *m/z* = 271.0968 (molecular formula calculated for C_I6_H_16_O_4_: 272.1049, difference 2.75 ppm).

*Gigantol* (**3**): *colorless gum,* UV (in MeOH) λ_max_ (log ξ) 206 (4.1), 281 (3) nm ^1^H-NMR (CDCl_3_, 400 MHz): δ = 6.22 (1H, d, *J* = 2.5 Hz, H-1), 6.21 (1H, d, *J* = 2.5 Hz, H-3), 6.66 (1H, dd, *J* = 8.3, 1.6 Hz, H-4a), 6.28 (1H, d, *J* = 1.6 Hz, H-4b), 6.81 (1H, d, *J* = 7.8 Hz, H-5), 6.61 (1H, d, *J* = 2 Hz, H-8), 2.79 (4H, m, H-9, H-10), 3.82 (3H, s, C5-OMe), 3.73 (3H, s, C7-OMe). For ^13^C NMR data (CDCl_3_, 100 MHz) δ =  107.6 (C-1), 156.1 (C-2), 98.0 (C-3), 160.4 (C-4), 120.5 (C-4a), 106.4 (C-4b), 114.0 (C-5), 144.1 (C-6), 145.8 (C-7), 110.7 (C-8), 133.8 (C-8a), 143.3 (C-10a), 37.2 (C-9), 38.2 (C-10), 55.4 (C-OMe), 54.8 (C-OMe); HR-ESI-MS ion [M-H]**^-^** at *m/z* = 273.1123 (molecular formula calculated for C_I6_H_18_O_4_: 274.1205, difference 3.56 ppm).

*Flavidin* (**4**): *colorless gum* UV (in MeOH) λ_max_ (log ξ) 214 (4.4) 286 (4.1), 300 (4.0) nm.^ 1^H-NMR (CD_3_OD, 400 MHz): δ = 6.30 (1H, d, *J* = 2.4 Hz, H-1), 6.19 (d, 1H, *J* = 2.4 Hz, H-3) 6.42 (1H, d, *J* = 2.2 Hz H-6), 6.55 (1H, d, *J* = 2.2 Hz H-8), 5.01 (2H, s, --O--CH_2_--Ar), and 2.78 (4H, s, H-9 and H-10).^13^C NMR data (CD_3_OD, 100 MHz) δ = 109.6 (C-1), 158.4 (C-2), 103.0 (C-3), 120.2 (C-4b), 113.0 (C-4a), 154.6 (C-4), 130.7 (C-5), 110.0 (C-6), 157.6 (C-7), 115.0 (C-8), 136.6 (C-8a), 135.3 (C-10a), 29.0 (C-9, 10), 69.4 (C- O--CH_2_--Ar). HR-ESI-MS ion [M+H] **^+^** at *m/z* = 241.0861 (Molecular formula calculated for C_15_H_12_O_3_:240.0786, difference 0.82 ppm).

*Coelonin* (**5**): *colorless gum* UV (in MeOH) λ_max_ (log ξ) 322 (4.6), 280 (4.3), 291 (4.2) nm^1^H-NMR (CD_3_OD, 400 MHz) δ = 6.31 (d, 1H, *J*  = 2.5Hz, H-1), 6.41 (1H, d, *J* = 2.5 Hz, H-3), 8 (1H, dd *J* = 7.2∶1.7 Hz, H-5), 6.62 (1H, dd *J* = 8∶2.1 Hz, H-6), 6.62 (1H, dd *J* = 8∶2.1 Hz, H-8), 2.63 (4H, s, H-9 and H-10), 3.80 (3H, s, C5-OMe). For ^13^C NMR data (CD_3_OD, 100 MHz) δ = 107.9 (C-1), 157.7 (C-2), 99.3 (C-3), 126.0 (C-4b), 117.0 (C-4a), 159.0 (C-4), 130.2 (C-5), 113.7 (C-6), 156.0 (C-7), 115.0 (C-8), 141.4 (C-10a), 140.6 (C-8a), 31.3 (C-10), 31.9 (C-9), 56.0 (C-OMe). HR-ESI-MS ion [M+H]^ +^ at *m/z* = 243.1022 (molecular formula calculated for C_I5_H_14_O_4_: 242.0943, difference 2.69 ppm).

δ = chemical shift in ppm

### Cell lines

HaCaT Keratinocytes were provided by LVMH recherche. Cells were cultivated in keratinocytes serum free medium (KSFM) complemented with 0.25% human recombinant EGF (Epidermal Growth Factor), 2.5% pituitary bovine extract, and 5% fetal bovine serum (FBS). All biological analyses were realized at passages 15 to 20.

### *In vitro* antioxidant analyses

All *in vitro* radical-scavenging experiments (DPPH, ^•^OH/luminol chemiluminescence) and IC_50_ calculations were assayed as previously described by Parejo *et al* [30]. Results were expressed as percentage inhibition of DPPH discolouration and luminol chemiluminescence reduction, respectively. The concentration effect (logarithmic curve) obtained for each compound was used to calculate IC_50_. Serial dilutions of **1**–**5**, and quercetin (used as standard reference) were prepared in MeOH.

### Analyses on HaCaT cells

For measurement of reactive oxygen species neutralization and PGE-2 quantification with *Vanda coerulea* stem biomarkers, HaCaT cells were seeded in black 96-well microplates (Greiner Bio One 655090) at a concentration of 10^3^ cells/well in complemented KSFM with 5% FBS. Microplates were incubated during 24 h (37°C, 5% CO_2_) before UV_B_ irradiation at 60 mJ/cm^2^ and treatment. For western blot analysis HaCaT cells were seeded at a density of 10^6^ cells/ 100 mm dishes and incubated during 48 h before irradiation with UV_B_ at 60 mJ/cm^2^ (UV Lamp, VL-6LM, Vilber Lourmat) and treatment. All experiments were performed at 90% cell confluency. Cell viability was previously evaluated thanks to XTT analysis according to Roehm NW *et al* [31]. Serial dilutions of **1**–**3**, were prepared in DMSO (0.1% v/v final concentration). The first higher concentration tested was not cytotoxic (data not shown).

### Reactive oxygen species neutralization on HaCaT

The DCFH-DA method was used to detect the levels of intracellular ROS [32] DCFH-DA diffuses into cells, where it is hydrolyzed by intracellular esterase to polar 2′,7′-dichlorodihydrofluorescein. This non-fluorescent fluorescein analog is oxidized by intracellular oxidants to a highly fluorescent, 2′,7′-dichlorofluorescein. Tocopherol [Sigma] (25 µM) was used as a positive reference. HaCaT cells were seeded as described and treated with serial dilutions of **1**–**3** and quercetin (standard reference) after 24 h incubation. Treated microplates were further incubated during 24 h (37°C, 5% CO_2_). HaCaT cells were washed with phosphate buffer saline (PBS), and DCFH-DA (0.05 mM) was added to the cells. 45 min after incubation, cells were stimulated by a H_2_O_2_ solution (100 µM in PBS) to generate an oxidative stress. Immediately after *F(T0)* and 40 min after stimulation *F(T40)*, DCFH fluorescence was detected at an excitation wavelength of 485 nm and an emission wavelength of 535 nm, using a SpectraFluor Plus spectrofluorometer (Tecan). Two kinds of assay controls were used: negative control was non-stimulated cells and positive control was H_2_O_2_ stressed cells (DMSO 0.1% v/v). Results were expressed in percentage of fluorescence inhibition (% of ROS neutralization) calculated by comparison with H_2_O_2_ stimulated cells.

(% of ROS neutralization)  =  [1- [*F(T40)-F(T0)]*_ sample_/*[F(T40)-F(T0)]*_positive control_] ×100.

Inhibition percentages were plotted according to compounds **1**–**3** and quercetin concentrations. Logarithmic regression analysis of the mean replicate values was used to calculate IC_50_ (Concentration causing half-maximal neutralization of ROS on H_2_O_2_ stressed HaCaT**)**

### Measurement of irradiated HaCaT PGE-2 production

Indomethacin (2.79 µM), a non-steroidal COX inhibitor, was used as a positive reference. Treated microplates were incubated at 37°C, 5% CO_2_. Untreated irradiated and non irradiated cells (DMSO 0.1% v/v final) were respectively used as positive and negative controls. After 24 h, supernatants were harvested for PGE-2 concentration measurement and BCA assay was realized to determine total cellular protein in each well. PGE-2 concentration was measured using the enzyme immunoassay Parameter™ PGE-2 kit (R&D Systems) according to the manufacturer's instructions. Each PGE-2 quantification (Q_PGE-2_) was reported to total protein concentration per well.

Results were expressed as inhibition percentage of PGE-2 (I PGE-2) production compared with untreated irradiated cells (positive control).

I PGE-2 = 100- ((Q_PGE2_) _sample_/ (Q_PGE2_) _positive control_) ×100.

Inhibition percentages were plotted according to compounds **1**–**3** concentrations. Logarithmic regression analysis of the mean replicate values was used to calculate IC_50_ (Concentration causing half-maximal Inhibition of irradiated HaCaT PGE-2 production**)**

### Measurement of COX-2 enzyme expression by Western blot

After UV_B_ irradiation 100 mm dishes were treated with stilbenoids **1**–**3** at 23 µM and 2.87 µM in a serum free medium and incubated during 16 h (37°C, 5% CO_2_). Untreated irradiated (UV_B_+) and non irradiated (UV_B_-) cells were used as positive and negative controls respectively. Total cell lysates were obtained using lysis buffer containing 0.5% SDS, 1% NP-40, 0.5% sodium deoxycholate, 150 mM NaCl, 50 mM Tris-HCl (pH 8) and proteases inhibitors. The protein concentration of each sample was determined using a Bradford assay. 30 µg of total protein was separated on a 10% polyacrylamide gel. Positive control CCD-1064SK cell lysate containing both β-actin and COX-2 was also loaded. The separated proteins were transferred to a nitrocellulose membrane. After blocking with 3% bovine serum albumin (BSA) in Tris-buffered-saline (TBS), the membranes were incubated with primary antibodies anti-COX-2 and anti-β-actin at a concentration of 1∶1000 in a 1% BSA TBS-T solution (containing 0.05% Tween 20) overnight at 4°C. Blot were washed twice with TBS-T and then incubated with a 1∶20000 dilution of horseradish peroxidase-conjugated secondary antibody for 1 h at room temperature. Blots were again washed three times with TBS and then developed using enhanced chemiluminescence (ECL) kit. The assay was repeated three times with similar results. Densitometry analyses were performed for each blot on the radiographic films using the ImageJ software. Results were expressed as fold decrease compared to the (UV_B_+) control. Each data was then reported to β-actin density.

### Measurement of COX-2 enzyme activity

Serial dilutions of **1**–**3** were prepared in DMSO. Six concentrations were tested from 0.1 µM to 100 µM for imbricatin (**1**), from 0.03 to 100 µM for methoxycoelonin **(2**) and from 0.3 µM to 100 µM for gigantol (**3**).

Stilbenoids **1**–**3**, indomethacin and water (negative control) were pre-incubated for 20 min at 22°C with the human recombinant COX-2 enzyme (0.2 µg) in buffer containing 100 mM Tris-HCl (pH 8.0), 2 mM phenol and 1 µM hematine. Thereafter, the reaction was initiated by addition of 2 µM arachidonic acid and the mixture was incubated for 5 min at 22°C. For basal control measurements, arachidonic acid was omitted from the medium. Indomethacin was used as positive inhibitory reference.

Following incubation, the reaction was stopped by the addition of 1 M HCl then 1 M Tris/HCl (pH 8.0) followed by cooling to 4°C. The amount of PGE-2 present in the reaction mixture was quantified using an EIA detection kit (Cayman Chemical).

The results were expressed as inhibition percentage of the control enzyme activity:

100–[(activity measured in the presence of the compound/control enzyme activity) ×100]

The IC_50_ values (concentration causing a half-maximal inhibition of control enzyme activity) and Hill coefficients (*nH*) were determined by non-linear regression analysis of the inhibition curves generated with mean replicate values using Hill equation curve fitting.

### Statistical Analysis

*In vitro* experiments were performed three times independently. All data were expressed as means ± standard deviations (sd). Treatment groups were compared by using one way analysis of variance ANOVA *post-hoc* test. Each stilbenoid effect was compared to positive control using student *t* tests. Statistical significance (*) was set at *p*<0.05.

### Supporting information

Confirmation of PGE-2 inhibitory effect on human normal epidermal keratinocytes is provided as **Figure S1**.

## Results and Discussion

Bio-guided fractionation performed on *Vanda coerulea* stems revealed that a stilbenoid-enriched CH_2_Cl_2_ extract displayed a better *in vitro* DPPH/ ^•^OH radical scavenging activity and inhibition of PGE-2 release from (UV_B_ 60 mJ/cm^2^) irradiated HaCaT than crude hydro-alcoholic extract. These observations led us to the isolation and identification of the five stilbenoids described in **Figure 1**. These stilbenoids are specific from the Orchidaceae family and were already described in other orchid genera [19], [20]. Imbricatin (**1**) was first identified in the orchid *Pholidota imbricata* [24]. It was found together with flavidin (**4**) in *Bulbophyllum* [19], *Coelogyne* and *Pholidota* species [24]–[26] and was also isolated with methoxycoelonin (**2**) in *Agrostophyllum callosum* and in *Coelogyne flaccida* [26]. Coelonin (**5**) was isolated from two species of *Coelogyne*: *C*. *ochracea* and *C. elata* [27]. Finally, the presence of gigantol (**3**) was identified for the first time in *Cymbidium giganteum* [28], and was also found together with coelonin (**5**) and methoxycoelonin (**2**) in *Cymbidium aloifolium* [29].

A comparison of phytochemical profiles of root, stem and leaf crude extracts showed that stilbenoids **1**–**5** were only present in *Vanda coerulea* stems. The most concentrated (**1**–**3**) were considered as *V. coerulea* stem biomarkers.

### Antioxidant activities

Each stilbenoid isolated from *Vanda coerulea* stems was already described for its radical scavenging properties mostly concerning DPPH neutralization [33]–[35]. Zhang X *et al* had evaluated radical scavenging properties of gigantol (**3**) using the DPPH (IC_50_ 56.4 µM) and ORAC assays [33]. Wang J *et al* and Guo XY *et al* indicated that coelonin (**5**) and imbricatin (**1**) neutralized DPPH radical with IC_50_ of 16.7 and 8.8 µM, respectively [34], [35]. Finally, the global antioxidant activities of flavidin (**4**) were evaluated using different antioxidant assays, including DPPH and hydrogen peroxide neutralization (without IC_50_ calculation) [36]. However, the radical scavenging capacities of stilbenoids **1** to **5** were not compared so far. Here, we aimed to propose a structure-activity relationship (SAR) relative to DPPH and ^•^OH radical scavenging activities.

**Figure 2:**
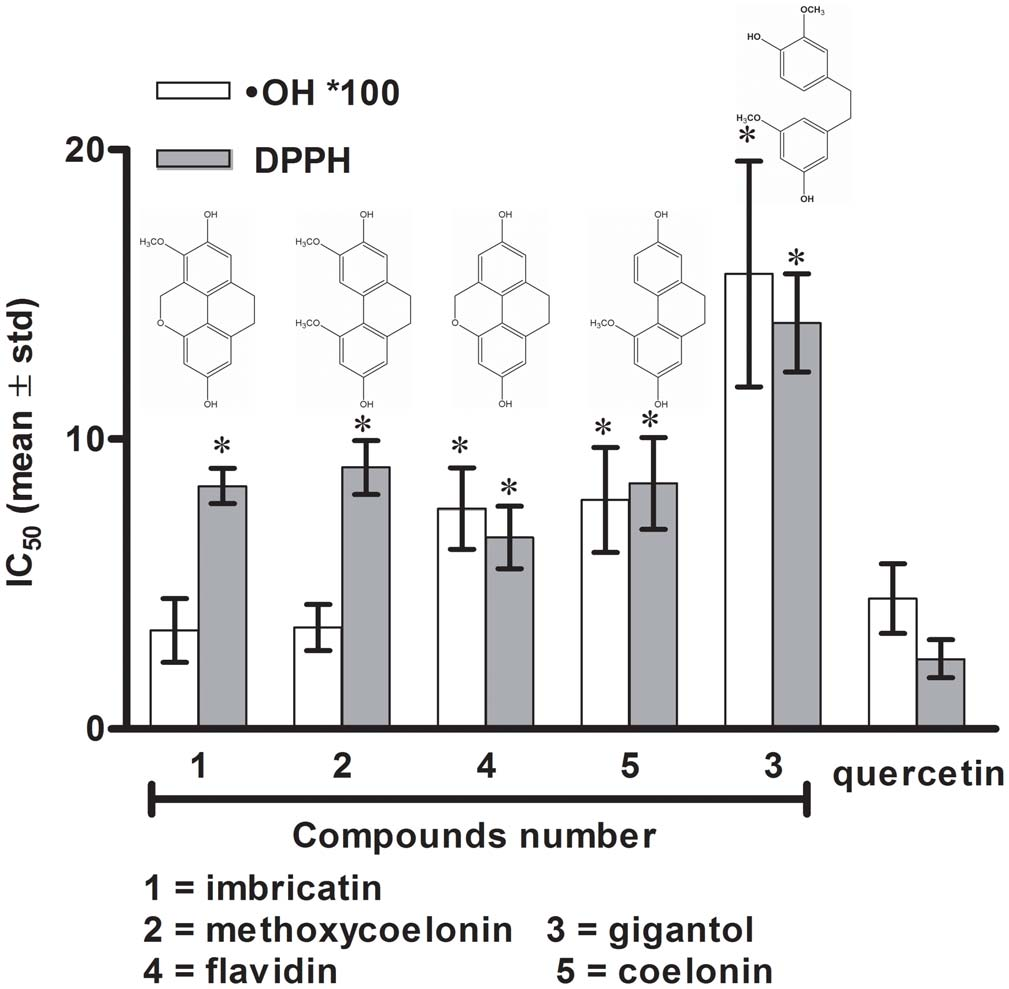
Radical scavenging activities of stilbenoids 1–5 on DPPH and OH radicals. Results are expressed as IC_50_ (mean ± sd) of stilbenoids determined on DPPH and ^•^OH radicals scavenging. IC_50_: concentration causing half-maximal neutralization of radical DPPH or ^•^OH. Quercetin was used as standard reference. IC_50_ values were compared to those obtained with the standard reference. Statistical significance (*) was set at *p*<0.05.

Among all the ROS produced,^ •^OH radicals are among the most aggressive forms implied in cellular oxidative stress [1]–[2]. Our results revealed that dihydro-phenanthropyrans (**1**, **4**) and dihydro-phenanthrenes (**2**, **5**) displayed better ^•^OH but also DPPH radicals scavenging properties than bibenzyle (**3**). Activities of imbricatin (**1**) and methoxycoelonin (**2**) were quite similar to the standard quercetin on ^•^OH radical scavenging activities, while flavidin (**4**) and coelonin (**5**) were globally less active. (**Figure 2** **,** **Table 1**).

**Table 1:** Radical scavenging activities of stilbenoids 1–5 on DDPH and ^•^OH radicals.

	IC_50_ (µM)	
Compounds	DPPH	^•^OH
Imbricatin (**1**)	8.37±0.61	0.034±0.011
Methoxycoelonin (**2**)	9.01±0.93	0.035±0.008
Gigantol (**3**)	14.01±1.69	0.157±0.039
Flavidin (**4**)	6.61±1.07	0.076±0.014
Coelonin (**5**)	8.47±1.58	0.079±0.018
Quercetin	2.42±0.65	0.045±0.012

Results are expressed as IC_50_ (mean ± sd) of stilbenoids **1**–**5** determined on DPPH and ^•^OH radical scavenging. IC_50_ concentration causing half-maximal neutralization of radical DPPH or ^•^OH. Quercetin was used as a standard reference.

SAR on ^•^OH radical scavenging indicated that dihydro-phenanthropyran and dihydro-phenanthrene structures had quite the same potentiality, which was enhanced by an *ortho*-methoxy-hydroxyl substitution on aromatic ring. According to these results, a classification could be proposed as followed (from the lowest to the highest IC_50_): **1**≈**2**>**4**≈**5**>**3** for ^•^OH radical scavenging activities.

We also observed a tendency for dihydro-phenanthropyran structures to better neutralize DPPH radical. Scavenging activities were better on structures without *ortho*-methoxy-hydroxyl substitution. Thus, a classification could be proposed as followed:

**4** >**1**≈**5**≥**2**>**3** for DPPH radical scavenging capacities.

In comparison with already published data, the IC_50_ of imbricatin (**1**) calculated for DPPH scavenging activities was in accordance with those indicated in literature [35].On the other side, better IC_50_ values were obtained with gigantol (**3**) and coelonin (**5**) than those previously described by Zhang X *et al* and Wang J *et al ,* respectively [33], [34].

The 9,10 dihydro-phenanthrenes (**2**, **5**) or phenanthropyrans (**1**, **4**) are planar structures compared with the bibenzyle gigantol (**3**). Thus, electron delocalization and structure stabilization is better on phenanthrenes than on bibenzyle. As explained by Jayaprakasha GK *et al*, the antioxidant capacities of 9,10 dihydro-phenanthrenes or phenanthropyrans may be attributed to hydrogen donating ability, the product phenoxyl radical being stabilized by resonance delocalization of the unpaired electron to the other position in the ring [36].

The excessive production of intracellular ROS plays an important role in the signalling events leading to gene activation [1]–[3]. Oxidative stress generated is responsible for a variety of conditions including skin ageing [2], [3], skin inflammation and cancer [1], [4]. Based on these premises, we examined whether the major stilbenoids (**1**–**3**), considered as *Vanda coerulea* stem biomarkers, could neutralize intracellular ROS in H_2_O_2_ stressed HaCaT cells.

**Figure 3:**
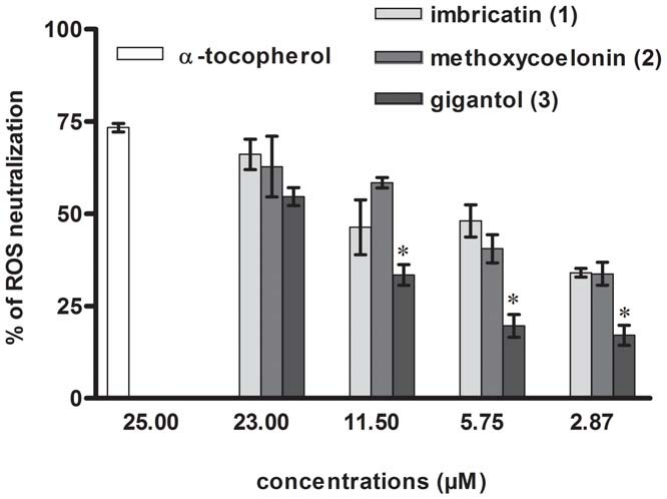
Reactive Oxygen Species (ROS) neutralization in H2O2 stressed HaCaT cells by stilbenoids 1–3. Results are expressed as percentages of total ROS neutralization in HaCaT cells calculated by comparison with the positive control (H2O2 stressed cell). Intracellular antioxidant properties of stilbenoids 1–3, considered as stem biomarkers, were evaluated on intracellular ROS neutralization. IC50 were calculated with more points than those represented on the graph. α-Tocopherol at 25 µM was used as a positive reference. (*) set at ***p***<0.05, gigantol (3) effect was statistically different from those of imbricatin (1) and methoxycoelonin (2).

The probe 2′,7′-dichlorofluorescin is used as an indicator of ROS formation in H_2_O_2_ (100 µM) stressed HaCaT. The intracellular fluorescence measurement using this probe mainly reflects the ability of stilbenoids to inhibit intracellular ^•^OH radical [37]. In this assay, we have compared the ROS scavenging activities of stem biomarkers. According to our previous cell viability analyses, the first higher concentration tested on HaCaT cells was not cytotoxic. Imbricatin (**1**) (IC_50_ 8.8 µM) and methoxycoelonin (**2**) (IC_50_ 9.4 µM) showed better ROS neutralization than gigantol (**3**) (IC_50_ 20.6 µM) and even than quercetin (IC_50_ 13.8 µM). (**Figure 3** **,** **Table 2**) These results were in accordance with our results obtained previously on ^•^OH radicals, with chemiluminescence. Consequently, we have demonstrated the intracellular ROS scavenging activities of these three stilbenoids on immortalized keratinocytes. Best intracellular antioxidant activities were obtained with the dihydro-phenanthropyran (**1**) and the dihydro-phenanthrene (**2**).

**Table 2:** Reactive Oxygen Species (ROS) neutralization in H_2_O_2_ stressed HaCaT cells.

Compounds	IC_50_ (µM)
Imbricatin (**1**)	8.8±1.7
Methoxycoelonin (**2**)	9.4±2.1
Gigantol (**3**)	20.6±0.5
Quercetin	13.8±2.2

Intracellular antioxidant property of stilbenoids **1**–**3** was evaluated on H_2_O_2_ stressed HaCaT by measuring ROS neutralization with DCFH-DA probe. Results are expressed as IC_50_ (mean ± sd) determined for global ROS neutralization on HaCaT. IC_50_ concentration causing half-maximal neutralization of intracellular ROS. Quercetin was used as a standard reference.

Intracellular ROS produced in excess contribute to inflammatory response triggered by UV radiations and have been suggested to be involved in UV_B_-induced COX-2 expression [1], [8]. In this study, we observed that stilbenoids (**1**–**3**) reduce the generation of intracellular ROS partly due to direct radical scavenging activities.

### Inhibition of PGE-2 production

PGE-2 is known to play an important role in UV-induced skin inflammation. Its production is mediated by UVs and ROS induction of COX-2 expression. Moreover, it has been reported that keratinocytes proliferation and apoptosis are also regulated by COX-2 after acute UV radiations [9].

**Figure 4:**
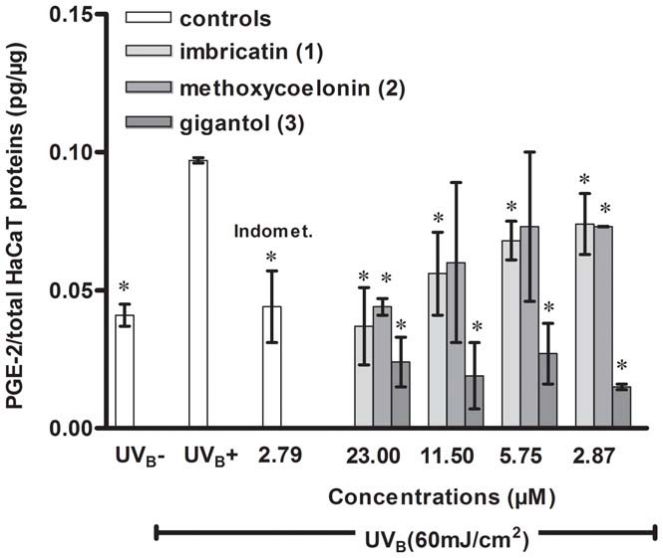
Concentration-dependent effect of stilbenoids 1–3 on PGE-2 release from irradiated (UVB 60 mJ/cm2) HaCaT. Histogram represents the quantity of PGE-2 produced (PGE-2/ total HaCaT proteins pg/µg) according to different treatments. Untreated irradiated (UVB+) and non irradiated (UVB-) HaCaT were used as positive and negative controls, respectively. Indomethacin (Indomet.) at 2.79 µM represented the positive reference (54% inhibition of PGE-2 production). Inhibition percentages of PGE-2 release were calculated by comparison with the positive control: UVB+. Values represent mean ± sd of three independent experiments. Treatment groups were compared by using one way analysis of variance ANOVA ***post-hoc*** test. Student paired ***t*** test was used to compare each stilbenoid effect to the positive control. Statistical significance (*) was set at ***p***<0.05. Significant differences were observed between each biomarker effect. IC50 were calculated with more points than those represented here on the graph.

Anti-inflammatory properties for gigantol (**3**) and coelonin (**5**) only, were already described in the scientific literature and referred mostly to the neutralization of nitric oxide production (and PGE-2 production for gigantol) on murine macrophages RAW 264.7 [38]–[40]. However, the skin anti-inflammatory properties of orchid stilbenoids have not been evaluated so far on HaCaT, a recognized cellular model [41], [42]. Therefore, we wanted to evaluate the potential of *V. coerulea* antioxidant stem biomarkers as skin anti-inflammatory agents. Their effects were evaluated on PGE-2 production and on inhibition of COX-2 expression in UV_B_ irradiated HaCaT cells. As shown in **Table 3** and **Figure 4**, the stilbenoids (**1**–**3**) inhibited PGE-2 release from UV_B_ (60 mJ/cm^2^) irradiated HaCaT cells. To compare biomarker activities the same range of concentrations was tested for each stilbenoid. Concentration-dependent effect was observed for imbricatin (**1**) (IC_50_ 12.2 µM) and methoxycoelonin (**2**) (IC_50_ 19.3 µM), but not for gigantol (**3**). The results revealed that even at the lowest concentration tested, gigantol **(3**) strongly inhibited PGE-2 production. We deduced from these data that these three major stilbenoids were principally responsible for the antioxidant and the reduction of PGE-2 production previously observed with *V. coerulea* crude stem extract. Bibenzyle gigantol (**3**) displayed better PGE-2 inhibition than dihydro-phenanthropyran (**1**) and dihydro-phenanthrene (**2**), but without any concentration-dependant effect at the tested concentrations.

**Table 3:** Effects of stilbenoids 1–3 on inhibition of HaCaT PGE-2 production and COX-2 enzyme activity.

	IC_50_ (µM)	
Compounds	PGE-2	COX-2
Imbricatin (**1**)	12.2±2.9	12.0±1.2
Methoxycoelonin (**2**)	19.3±3.8	5.8±1.2
Gigantol (**3**)	No concentration-dependanteffect	≥100
Indomethacin	2.7±0.7	1.6±0.4

Results are expressed as IC_50_ (mean ± sd) determined for inhibition of PGE-2 release from UV_B_ (60 mJ/cm^2^) irradiated HaCaT and inhibition of human recombinant COX-2 activity. IC_50_ concentration causing half-maximal inhibition of HaCaT PGE-2 production and concentration causing a half-maximal inhibition of COX-2 control specific activity. Gigantol demonstrated strong inhibition of PGE-2 production but did not display concentration dependent effect at the tested concentrations, thus IC_50_ could not be calculated. Indomethacin was used as a positive reference.

This global reduction of PGE-2 production could be attributed to an inhibition of COX-2 enzyme activity and/or an attenuation of UV_B_-induced COX-2 expression.

**Figure 5:**
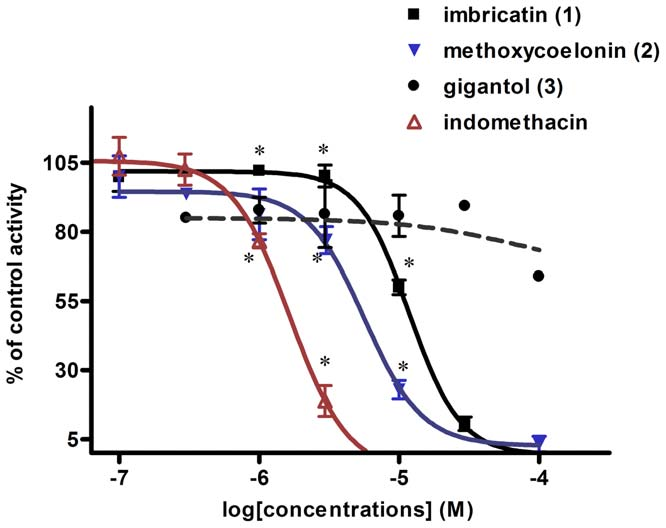
Inhibition of human recombinant COX-2 enzyme by stilbenoids 1–3. The effect of stilbenoids 1–3 was evaluated on human recombinant COX-2 activity by measuring the formation of PGE-2 from arachidonic acid. Results were expressed as percentage of control enzyme activity functions of stilbenoids 1–3 concentrations (log [concentration] mol/L). Experiments were carried out in triplicate. Indomethacin was used as a standard inhibitory reference. Statistical significance (*) was set at ***p***<0.05 compared to the standard reference. Imbricatin (1) and methoxycoelonin (2) inhibited significantly COX-2 enzyme activity. Gigantol (3) did not display significant inhibition of COX-2 enzyme. Significant differences were only observed at 10 µM between imbricatin (1) and methoxycoelonin (2).

Interestingly, (**Figure 5** **,** **Table 3**) imbricatin (**1**) and methoxycoelonin (**2**) but not gigantol (**3**) were able to inhibit human recombinant COX-2 enzyme (IC_50_ 12.0 µM and 5.8 µM, respectively), suggesting that dihydro-phenanthropyran (**1**) and dihydro-phenanthrene (**2**) but not bibenzyle (**3**) could reduce PGE-2 production through COX-2 inhibition. Their effect on human recombinant COX-2 enzyme was comparable to the activity of indomethacin (IC_50_ 1.6 µM), a classical non-steroidal COX inhibitor.

The IC_50_ calculated for inhibition of PGE-2 production on HaCaT are different from those calculated for COX-2 inhibition. Indeed, for the first analysis we have measured stilbenoid activities on the whole cell, determining a global response leading to the release of PGE-2. For the second analysis, we have directly determined stilbenoids effects on human recombinant and isolated COX-2 enzyme. Because of these differences between cellular and enzymatic analysis the IC_50_ calculated in both experiments were not identical.

Wong J.H. *et al* 2006 have demonstrated that gigantol (**3**) was able to inhibit PGE-2 production on murine macrophages RAW 264.7 as a result of inhibition of COX-2 expression but also of NF-κB expression [38]. We hypothesized that the constant inhibition effect of (**3**) on HaCaT PGE-2 production could be explained by a strong reduction of UV_B_-induced COX-2 enzyme expression.

**Figure 6:**
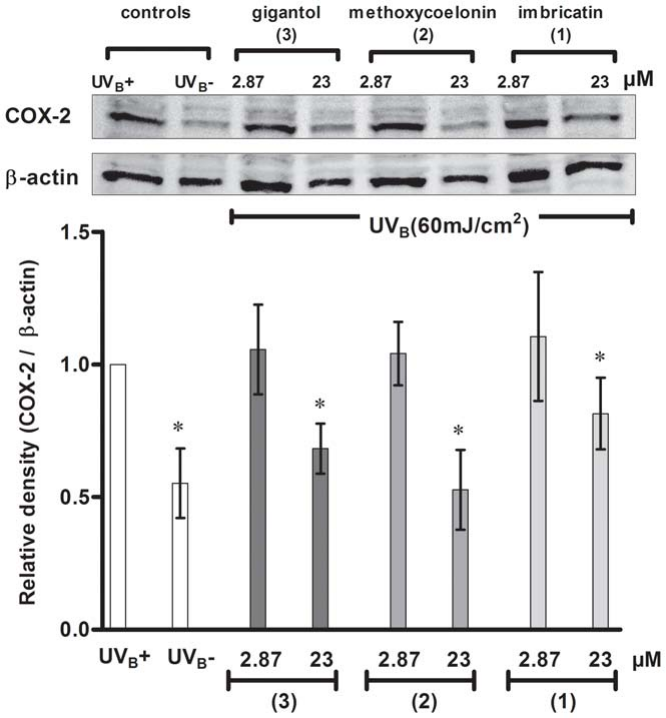
Effect of stilbenoids 1–3 on COX-2 expression in irradiated HaCaT cells. The effect of stilbenoids 1–3 was evaluated on COX-2 expression on HaCaT cells after UVB (60 mJ/cm2) irradiations. Untreated irradiated (UVB+) and non irradiated (UVB-) HaCaT were respectively used as positive and negative controls. In bar graph, the relative intensity of COX-2 was expressed as a ratio of COX-2/β-Actin and compared to the positive control UVB+. Statistical significance (*) was set at ***p***<0.05 compared to the positive control. The three stilbenoids inhibited UVB-induced COX-2 expression at 23 µM, but did not inhibit COX-2 expression at 2.87 µM. Experiment was performed in triplicate with similar results.

Western blot analysis was performed with UV_B_ (60 mJ/cm^2^) irradiated HaCaT cell lysates. Two concentrations of each stilbenoids were tested (23 and 2.87 µM) on HaCaT. As shown in **Figure 6**, the three biomarkers were able to attenuate UV_B_-induced COX-2 expression at 23 µM. Best results were obtained with methoxycoelonin (**2**) and gigantol (**3**). They did not inhibit COX-2 expression at the lowest concentration tested (2.87 µM). Gigantol demonstrated strong inhibition of PGE-2 production even at 2.87 µM but did not inhibit COX-2 expression and activity at this concentration. Gigantol (**3**) may therefore act through other mechanisms to exert its inhibitory effect on PGE-2 production and COX-2 enzyme may not be its only biological target.

UV_B_ exposure induces ROS accumulation in human skin and is involved in its ageing process as well as its inflammatory response [1]–[3]. This accumulation would explain a higher induction of COX-2 and consequently a higher PGE-2 release [5]. Irradiated HaCaT cells provide a good model to measure PGE-2 production and allowed the evaluation of the potential keratinocytes anti-inflammatory properties of the major stilbenoids isolated from *V. coerulea* stems. Our results revealed that the dihydro-phenanthrene methoxycoelonin (**2**) and the dihydro-phenanthropyran imbricatin (**1**) displayed the best intracellular antioxidant activities on HaCaT keratinocytes and interesting anti-inflammatory properties due to a concentration-dependant inhibition of COX-2 activity and expression. The bibenzyle gigantol (**3**) better inhibited HaCaT PGE-2 production compared to methoxycoelonin (**2**) and imbricatin (**1**) at each concentration tested. Its activity could be partly attributed to an attenuation of UV_B_-induced COX-2 expression. No concentration-dependent inhibitory effect was observed to allow IC_50_ calculation for gigantol (**3**) effect on PGE-2 production. Wong JH *et al* also demonstrated that this compound (**3**) was able to reduce NF-κB expression on murine macrophages RAW 264.7 [38]. Thus, this transcriptor factor could be another target, leading to a reduction of PGE-2 synthesis and to a global anti-inflammatory effect on irradiated HaCaT. Interestingly, our results revealed that gigantol (**3**) displayed a 16-fold stronger inhibition of PGE-2 production on human HaCaT than on murine macrophage RAW 264.7 [38].

In summary, major stilbenoids (**1**–**3**), considered as *Vanda coerulea* stem biomarkers, provided chromophore structures absorbing UV_B_ radiations. Applied locally these polyphenolic compounds may act as a sunscreen [11]. Our data *hitherto* obtained showed that imbricatin (**1**), methoxycoelonin (**2**) and gigantol (**3**) displayed antioxidant properties on HaCaT by means of intracellular ROS neutralization. The three stilbenoids were able to reduce UV_B_-induced COX-2 expression, thereby reducing PGE-2 production on HaCaT keratinocytes. These activities could be partly linked to the reduction of intracellular oxidative stress, to a reduction of COX-2 expression and, for imbricatin (**1**) and methoxycoelonin (**2**) to a direct inhibition of COX-2 enzyme activity. Globally, we observed complementary properties between the dihydro-phenanthropyran imbricatin (**1**) the dihydro-phenanthrene methoxycoelonin (**2**) and the bibenzyle gigantol (**3**). The first displayed better antioxidant activities whereas the latter showed strong inhibition of PGE-2 production. Anti-inflammatory property of orchid's stilbenoids was here described for the first time on HaCaT keratinocytes. *Vanda coerulea* stem biomarkers could be considered as potential new natural skin photoprotecting agents.

## Supporting Information

Figure S1 Concentration-dependent effect of stilbenoids (1-3) on PGE-2 release from irradiated (UVB 60 mJ/cm2) normal human epidermal keratinocytes (NHEK). Histogram represents the quantity of PGE-2 produced (PGE-2/ total NHEK proteins pg/µg) according to different treatments. Untreated irradiated (UVB+) and non irradiated (UVB-) NHEK were used as positive and negative controls, respectively. Indomethacin (Indomet.) at 2.79 µM represented the positive reference (86% inhibition of PGE-2 production). Inhibition percentages of PGE-2 release were calculated by comparison with the positive control (UVB+). Values represen mean ± sds calculated with three independent experiments. Treatment groups were compared by using one way analysis of variance ANOVA post-hoc test. Student paired t test was used to compare each stilbenoid effect to the positive control. Statistical significance (*) was set at p<0.05. Significant differences were observed between each biomarker effect. IC50 were calculated with more points than those represented here on the graph.
(0.82 MB TIF)
