# Supplementary figures and images for: Antioxidant Biomarkers from Vanda coerulea Stems Reduce Irradiated HaCaT PGE-2 Production as a Result of COX-2 Inhibition
Source: PLoS One. 2010 Oct 28;5(10):e13713. doi: 10.1371/journal.pone.0013713 (PMC2965657; doi:10.1371/journal.pone.0013713)

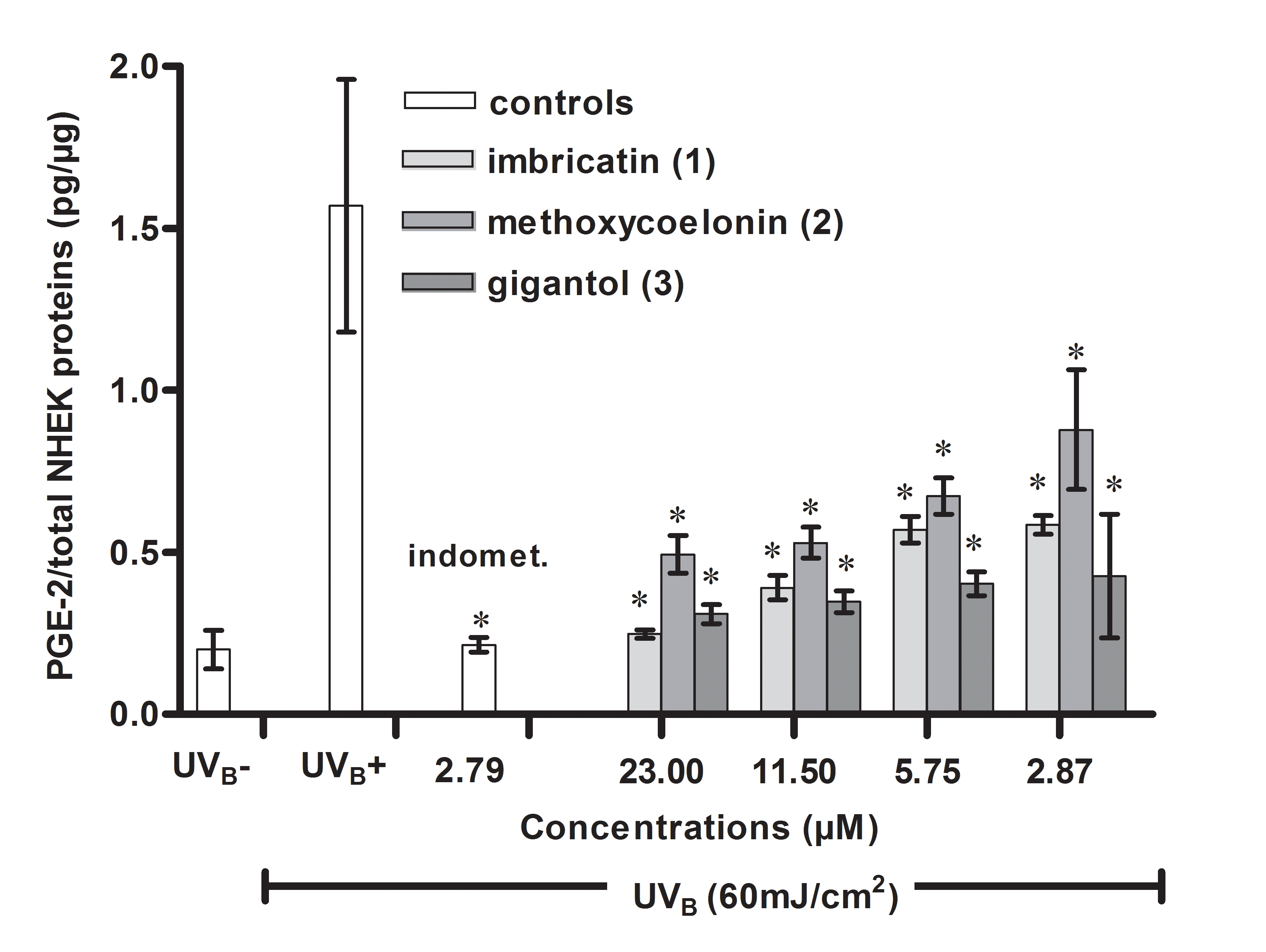

Supplement: Figure S1 — Concentration-dependent effect of stilbenoids (1-3) on PGE-2 release from irradiated (UVB 60 mJ/cm2) normal human epidermal keratinocytes (NHEK). Histogram represents the quantity of PGE-2 produced (PGE-2/ total NHEK proteins pg/µg) according to different treatments. Untreated irradiated (UVB+) and non irradiated (UVB-) NHEK were used as positive and negative controls, respectively. Indomethacin (Indomet.) at 2.79 µM represented the positive reference (86% inhibition of PGE-2 production). Inhibition percentages of PGE-2 release were calculated by comparison with the positive control (UVB+). Values represen mean ± sds calculated with three independent experiments. Treatment groups were compared by using one way analysis of variance ANOVA post-hoc test. Student paired t test was used to compare each stilbenoid effect to the positive control. Statistical significance (*) was set at p<0.05. Significant differences were observed between each biomarker effect. IC50 were calculated with more points than those represented here on the graph. (0.82 MB TIF) [file pone.0013713.s001.tif]
